# Genome-Wide SNP Analysis Reveals Population Structure and Genetic Diversity in *Lycium ruthenicum* Murr

**DOI:** 10.3390/plants14213374

**Published:** 2025-11-04

**Authors:** Rong Yang, Xiuhua Wu, Yu’e Bai, Yujiao He, Sujuan Chang, Long Hai

**Affiliations:** 1 Inner Mongolia Academy of Forestry Sciences, Hohhot 010010, China; yangrong123@emails.imau.edu.cn (R.Y.);; 2 Inner Mongolia Engineering Technology Research Center of *Lycium ruthenicum* Murr., Hohhot 010010, China; 3 Key Laboratory of State Forestry and Grassland Administration for Sandy Land Biological Resources Conservation and Cultivation, Hohhot 010010, China; 4 College of Forestry, Inner Mongolia Agricultural University, Hohhot 010019, China

**Keywords:** SLAF-seq, germplasm resources, genetic diversity, population genomics, single nucleotide polymorphism

## Abstract

*Lycium ruthenicum* Murr. (Black goji), a medicinal and economically valuable crop rich in bioactive compounds, remains genomically understudied despite its expanding cultivation. To overcome limitations of traditional markers in genetic diversity analysis and molecular breeding, we employed specific-locus amplified fragment sequencing (SLAF-seq) to develop genome-wide SNP markers and elucidate the genetic structure of 213 *L. ruthenicum* accessions from natural and cultivated populations in Alxa, China. We identified 827,630 SLAF tags and 33,121 high-quality SNPs uniformly distributed across 12 chromosomes, establishing the first high-density SNP database for this species. Population genetic analyses revealed three distinct genetic clusters with <60% geographic origin consistency, indicating weakened isolation due to anthropogenic germplasm exchange. The Qinghai Nuomuhong population exhibited the highest genetic diversity (Nei’s index = 0.253; Shannon’s index = 0.352), while low overall polymorphism (average PIC = 0.183) likely reflects SNP biallelic limitations and domestication bottlenecks. Notably, SNP-based clustering showed <40% concordance with phenotypic trait clustering (31 traits), underscoring environmental plasticity as a key driver of morphological variation. This study provides the first genome-wide SNP resource for *L. ruthenicum*, enabling marker-assisted breeding and highlighting the need for standardized germplasm management to mitigate genetic erosion.

## 1. Introduction

*Lycium ruthenicum* Murr. (black goji), a medicinally and economically significant species in the Solanaceae family, has experienced growing demand for functional foods and pharmaceutical applications due to its fruit’s high anthocyanin, polysaccharide, and antioxidant content [1]. Despite this increasing cultivation, genomic research on *L. ruthenicum* lags behind that of its close relative *L. barbarum* (wolfberry), with fundamental aspects of its genetic diversity, population evolutionary relationships, and key trait regulation remaining poorly characterized [2]. Current cultivars predominantly derive from direct domestication of wild populations or phenotypic selection, yet the absence of a robust molecular marker system has resulted in widespread cultivar misidentification and synonymy, significantly impeding germplasm characterization and molecular breeding efforts [3]. While traditional morphological markers and low-throughput molecular markers (e.g., SSR, ISSR) can support basic genetic analyses, their limited density and uneven genomic distribution hinder the investigation of complex traits influenced by polygenic interactions or genotype environment effects. This limitation is evident in studies where markers such as SSRs, despite revealing population structure, show constrained power in dissecting polygenic traits like drought tolerance in rice [4], or where different marker systems (e.g., ISSR, CDDP, SCoT) yield varying estimates of genetic diversity and differentiation within barley populations, underscoring the need for more comprehensive genomic approaches [5]. Consequently, developing high-throughput molecular markers and constructing a comprehensive genetic map for *L. ruthenicum* is essential for advancing germplasm improvement.

Among reduced-representation sequencing approaches, specific-locus amplified fragment sequencing (SLAF-seq) has emerged as a powerful tool for large-scale single nucleotide polymorphism (SNP) discovery. This technique targets restriction-enzyme-digested genomic regions, offering an optimal balance of cost efficiency and marker density [6]. The high-throughput nature of SNP markers has revolutionized genetic map construction, identification of phenotype-associated loci, and marker-assisted breeding [7]. Successful applications span diverse crops including maize (*Zea mays*) [8], rice (*Oryza sativa*) [9], and soybean (*Glycine max*) [10]. For example, Yu et al. [11] combined SLAF-seq with bulked segregant analysis (BSA) in an F_2_ population, identifying four chromosomal regions (totaling 25.71 Mb) containing 1513 annotated genes associated with low-temperature germination tolerance. Similarly, Ren et al. [12] developed a high-density genetic map (8078 markers spanning 3780.98 cM) using an F_6_:_9_ recombinant inbred line population, revealing 23 drought-related QTLs with three co-localized loci on chromosomes 6, 17, and 19.

Recent applications have extended SLAF-seq to woody and medicinal plants, including studies of gene expression patterns in early-budding tea (*Camellia sinensis*) [13] and population genetic analyses of Chinese honeysuckle (*Lonicera japonica*) germplasm. While SLAF-seq has proven valuable for QTL mapping in *L. barbarum* [14,15] and enabled construction of ultra-dense genetic maps [16], comprehensive SNP development and population genetic studies in *L. ruthenicum* remain lacking. This gap persists despite existing resources like global wolfberry germplasm databases [17] and Romanian *Lycium* genotyping data [18], which are yet to incorporate SLAF-seq. Although SSR markers have addressed some limitations of traditional approaches [19], their moderate polymorphism (PIC 0.612–0.911) remains inadequate for resolving complex traits in *L. ruthenicum*.

In this study, we employ SLAF-seq for genome-wide SNP discovery in *L. ruthenicum*, analyzing 213 germplasm accessions from Alxa League, Inner Mongolia. Our objectives are threefold: (1) to establish a high-density SNP marker database addressing current limitations of low-polymorphism markers; (2) to elucidate genetic structure differences between wild and cultivated populations, clarifying domestication impacts on diversity; (3) to identify candidate loci associated with phenotypic traits to facilitate marker-assisted breeding. This work addresses critical gaps in *Lycium* genomics and provides essential technical support for germplasm conservation, cultivar identification, and molecular breeding in *L. ruthenicum*.

## 2. Materials and Methods

### 2.1. Plant Materials

A total of 213 *L*. *ruthenicum* accessions were collected from the germplasm repository in Alxa Left Banner, Inner Mongolia (Table 1). Fresh and healthy young leaves from each accession were wrapped in pre-labeled aluminum foil, flash-frozen in liquid nitrogen, and stored at −80 °C for subsequent DNA extraction.

### 2.2. Genomic DNA Extraction

Genomic DNA was isolated using a modified CTAB method [20]. Briefly, leaf tissues were ground in liquid nitrogen-precooled steel beads using a homogenizer (Scilogex, CF1524R, Rocky Hill, CT, USA) at 1500 rpm for 40 s, followed by incubation in preheated CTAB lysis buffer (Tiangen, DP1403, Beijing, China) containing 2% β-mercaptoethanol at 65 °C for 40–60 min. Protein contaminants were removed by two rounds of chloroform/isoamyl alcohol (24:1) extraction (12,000 rpm, 20 min). DNA was precipitated by adding isopropanol and 3 M sodium acetate (10:1 v/v) at −20 °C for 1 h, washed with 75% ethanol (12,000 rpm, 5 min), air-dried, and dissolved in RNase A-treated ddH_2_O. DNA integrity was verified via 1% agarose gel electrophoresis (using λ-Hind III digest DNA Marker, Takara, Dalian, China) at 120 V for 30 min, and purity was assessed using a spectrophotometer (A_260_/A_280_ = 1.8–2.0). Qualified samples were diluted to 18 ng/μL and stored at −20 °C.

### 2.3. SLAF-Seq Library Construction and Sequencing

#### 2.3.1. Reference Genome and Restriction Enzyme Selection

SLAF-seq libraries were constructed for 213 *L. ruthenicum* DNA samples following a modified protocol [21]. The *Lycium chinense* genome was selected as the reference for in silico restriction enzyme prediction [22], considering genome size, GC content, and fragment distribution. Enzyme selection criteria included: (1) low fragment duplication rates, particularly in repetitive regions; (2) uniform genomic distribution of digested fragments; (3) compatibility between experimental conditions and predicted fragment lengths [23]; (4) optimal SLAF tag yield for downstream analysis. The optimal restriction enzyme combination was selected, and genomic DNA was digested accordingly.

#### 2.3.2. SLAF-Seq Sequencing

**Figure 1:**
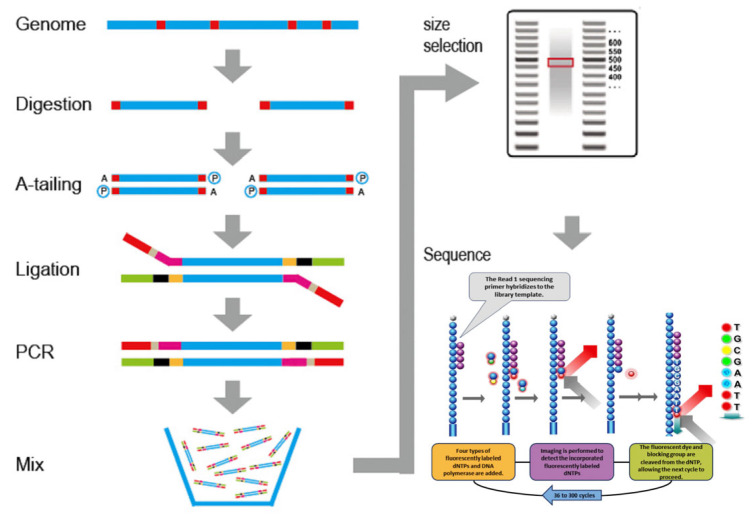
The process of SLAF-seq technology.

The SLAF-seq workflow is illustrated in Figure 1. Briefly, digested DNA fragments were A-tailed, ligated with dual-index adapters, and PCR-amplified. Amplified products were size-selected via 2% agarose gel electrophoresis, purified, and sequenced on the Illumina HiSeq2500 platform.

### 2.4. Data Processing and SNP Discovery

Raw sequencing reads were demultiplexed using dual-index barcodes. Low-quality reads (Q30 < 90%, adapter contamination, or abnormal GC content) were filtered out. Digestion efficiency was evaluated by comparing experimental and in silico digestions.

**Figure 2:**
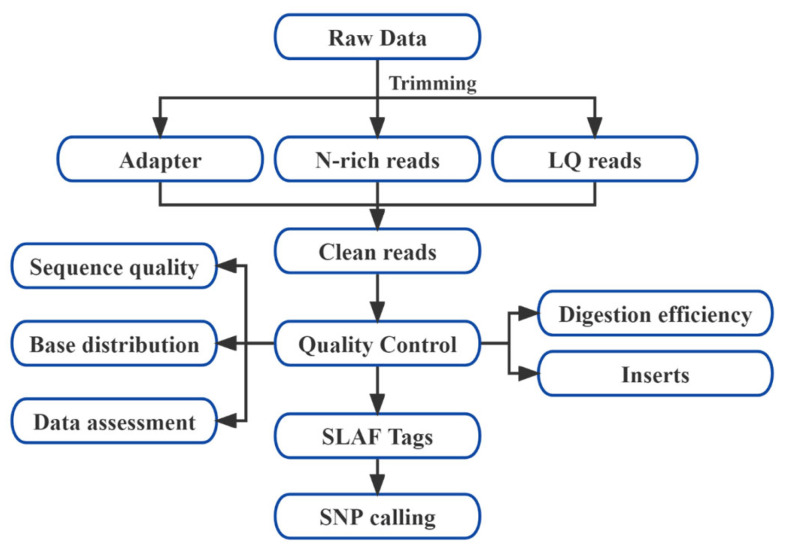
The development process of SNP marker.

Clean reads were aligned to the *L. chinense* reference genome using BWA v0.7.17 [24]. SNPs were called using both Samtools v1.9 [25] and GATK v3.8 [26], with high-confidence SNPs retained from the intersection of both datasets (Figure 2).

### 2.5. Population Genetic Structure Analysis

Population genetic studies investigate the frequencies of genes and genotypes within populations, as well as the factors influencing these frequencies. High-confidence SNP loci were obtained by filtering variant SNP data based on minor allele frequency (MAF ≥ 0.05) and locus integrity (INT ≥ 0.3). These high-quality SNPs were subsequently used for downstream analyses, including phylogenetic tree construction, population structure analysis, principal component analysis (PCA), and linkage disequilibrium (LD) analysis [27]. In this study, population structure was analyzed using ADMIXTURE software (version 1.22) [28]. Phylogenetic relationships were inferred using MEGA X [29] with the neighbor-joining algorithm [30]. PCA was performed using EIGENSOFT (version 6.0) [31] to assess sample clustering patterns, providing additional insights into evolutionary relationships.

### 2.6. Genetic Diversity Analysis

Genetic diversity was assessed using multiple approaches. Kinship coefficients were calculated using GCTA (version 1.92.1) [32], while genetic distances were computed using MEGA X [29]. Custom Perl scripts developed by Biomarker Technologies were employed to analyze key genetic diversity indices, including minor allele frequency (MAF), expected number of alleles (Ne), expected heterozygosity (He), Nei’s diversity index (H), number of polymorphic loci (A), observed number of alleles (Na), observed heterozygosity (Ho), polymorphism information content (PIC), and Shannon-Wiener index (I). These analyses provided a comprehensive evaluation of genetic diversity within the *L. ruthenicum* population.

### 2.7. Construction of L. ruthenicum DNA Fingerprints

SNP markers were developed using SLAF-seq data from 213 *L. ruthenicum* accessions. The selection criteria included uniform genomic distribution of markers, no missing data (100% locus integrity), MAF ≥ 0.20, PIC ≥ 0.35, Hardy–Weinberg equilibrium (HWE) *p*-value > 0.01, and no adjacent mutations within 100 bp flanking regions. High-confidence SNPs meeting these criteria were designated as core markers. These were subsequently converted into QR codes to establish a standardized *L. ruthenicum* DNA fingerprinting database.

## 3. Results and Analysis

### 3.1. Restriction Enzyme Selection and Library Construction Evaluation

**Figure 3:**
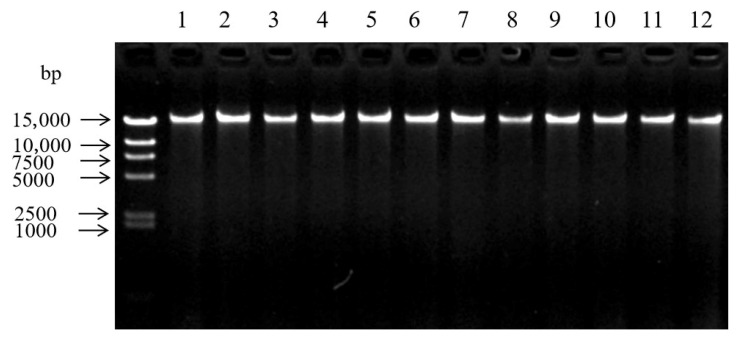
The detection of DNA extraction of B1~B12 *L. ruthenicum* leaves.

Through in silico restriction enzyme prediction analysis, *Hae*III and *Hpy*166II were identified as the optimal restriction enzymes for genomic digestion. Genomic DNA from all 213 *L. ruthenicum* samples that passed quality control was subjected to digestion with these enzymes. As shown in Figure 3 (samples B1–B12), agarose gel electrophoresis confirmed high DNA extraction quality across all samples.

**Figure 4:**
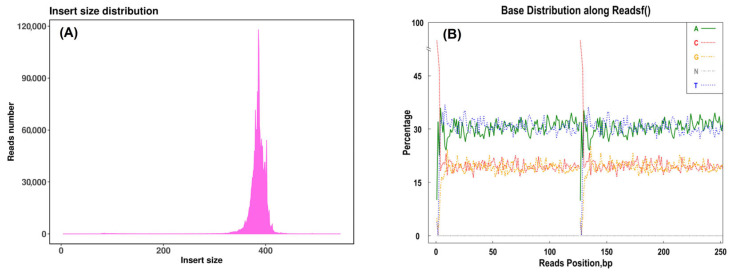
The reference sequence insert size distribution and 4 bases content distribution. (**A**) displays the insert size distribution of control sequences, while (**B**) shows the nucleotide base composition. The first 126 bp represent the base distribution of Read 1 in paired-end sequencing, and the subsequent 126 bp represent Read 2, with each base position (bp) corresponding to an individual sequenced nucleotide.

Post-digestion bioinformatics analysis mapped paired-end sequences to the reference genome to determine SLAF tag lengths. The insert size distribution (Figure 4A) revealed fragments ranging from 364 to 394 bp, following a normal distribution, indicating successful SLAF library construction. Base composition analysis (Figure 4B) showed balanced A/T and C/G distributions, confirming sequencing accuracy and reliability of the SLAF tags for subsequent SNP development and genetic diversity analysis.

### 3.2. Statistical Analysis and Quality Assessment of Reduced-Representation Genome Sequencing

**Figure 5:**
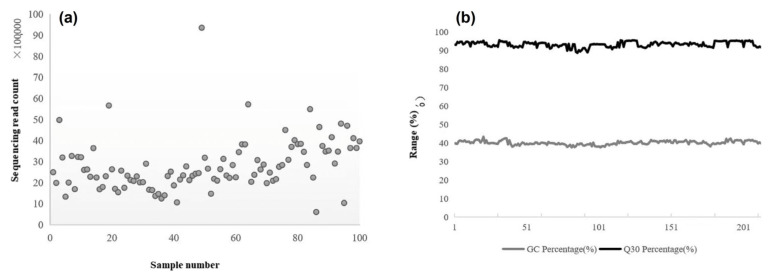
Sequencing statistics showing (**a**) total reads per sample and (**b**) population-level GC content and Q30 scores distribution.

To ensure data quality for downstream analyses, we employed 126 bp paired-end sequencing based on the results shown in Figure 5. Sequencing of 213 *L. ruthenicum* accessions generated a total of 563.48 million reads, with individual samples yielding between 606,309 (ES14 from Ejin Banner’s Suobaoerge Sumu) and 9,355,410 reads (E11 from Dalaihubu Town, Ejin Banner) (Figure 5a).

Quality assessment revealed Q30 scores ranging from 88.68% to 95.55%, with an average of 93.32% across all samples. The sample with lowest read count (ES14) showed the minimum Q30 value (88.68%), while BH11 from Nuomuhong, Qinghai Province exhibited the highest quality score (95.55%). Notably, all samples exceeded the 90% Q30 threshold, confirming data reliability (Figure 5a). GC content analysis demonstrated values between 37.62% (ED6 from Dongfeng Town, Ejin Banner) and 43.37% (B29 from Urad Rear Banner, Bayannur), with a population average of 40.11%, meeting standard sequencing requirements (Figure 5b).

### 3.3. Development of SLAF Tags and SNP Markers

**Figure 6:**
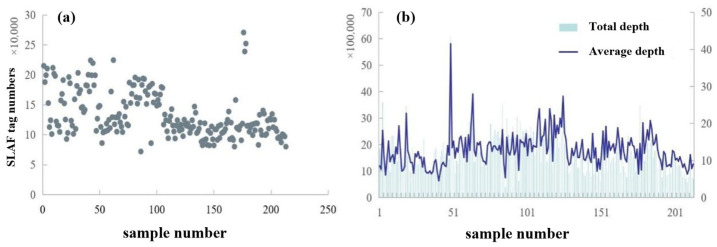
SLAF tag statistics showing (**a**) SLAF tag counts and (**b**) total sequencing depth and average depth per sample.

Sequencing analysis of 213 *L. ruthenicum* accessions yielded 827,630 high-quality SLAF tags, with individual samples containing between 72,074 and 270,676 tags (Figure 6a). The population exhibited an average total sequencing depth of 1,692,421× and mean depth of 12.86×, with per-sample values ranging from 389,796–6,076,553× and 5.4–41.5×, respectively (Figure 6b).

**Figure 7:**
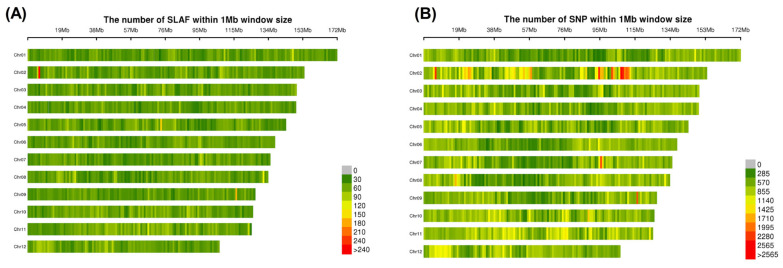
Chromosomal distribution of SLAF tags and SNPs in *L. ruthenicum*: (**A**) Density of SLAF tags; (**B**) Density of genome-wide SNPs.

Alignment using BWA software (0.7.10-r789) mapped the SLAF tags to the *L. chinense* reference genome, identifying 35,098 polymorphic SLAF tags distributed across chromosomes (Table 2). Intersection analysis of SNP calls from both GATK (GenomeAnalysis Toolkit, v4.1) and Samtools (v1.12) pipelines generated a robust dataset of 1,135,505 SNPs. Stringent filtering based on locus integrity (INT ≥ 0.3) and minor allele frequency (MAF ≥ 0.05) produced 33,121 high-confidence SNPs. Chromosomal distribution analysis revealed uniform dispersion of both polymorphic SLAF tags and SNPs across all 12 chromosomes (Figure 7), confirming data quality for subsequent analyses.

### 3.4. Phylogenetic Analysis of L. ruthenicum

**Figure 8:**
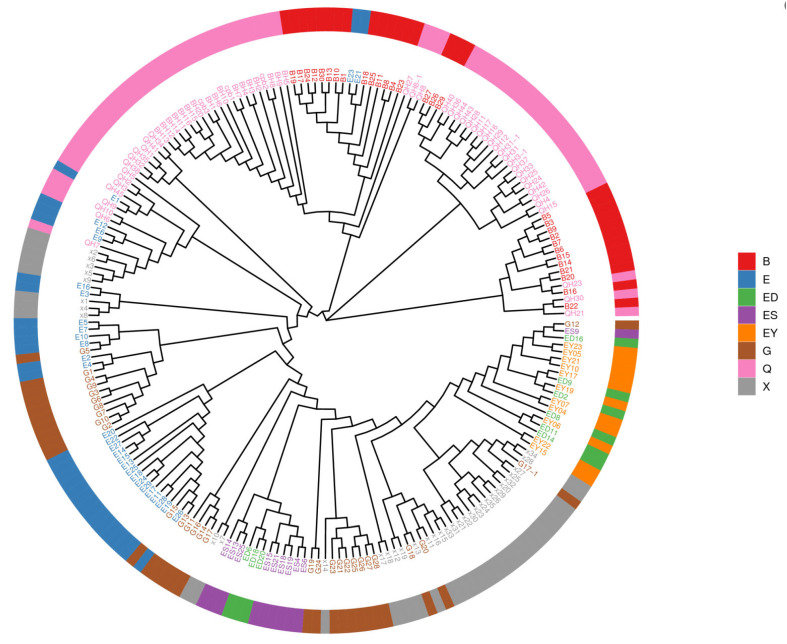
Phylogenetic tree of 213 *L. ruthenicum* based on SNPs. The neighbor-joining tree, constructed based on genome-wide SNP data, reveals three distinct genetic clusters (G1, G2, and G3). The clustering pattern shows a weak correlation with geographic origins, indicating extensive germplasm exchange and genetic admixture among populations from different regions.

Phylogenetic trees were constructed to elucidate evolutionary relationships among the 213 *L. ruthenicum* accessions from eight geographic origins. Using MEGA X software with the neighbor-joining method, we generated a tree based on *p*-distance matrices with 1000 bootstrap replicates (Figure 8, Table 3). The analysis revealed three distinct clades (G1, G2, and G3), each containing further subdivisions.

Group 1 (G1) comprised 39 accessions (18.31%), predominantly from Nuomuhong County, Qinghai Province (23 accessions) and Urad Rear Banner, Bayannur City, Inner Mongolia (14 accessions), suggesting shared ancestry between these populations. Group 2 (G2) contained 39 accessions (18.31%), with 24 from Nuomuhong County and 15 from Urad Rear Banner. Group 3 (G3), the largest cluster (63.38%), included 135 accessions from seven locations across four provinces. Notably, accessions did not cluster strictly by geographic origin, reflecting substantial genetic variation and diversity within the species.

### 3.5. Population Structure Analysis

**Figure 9:**
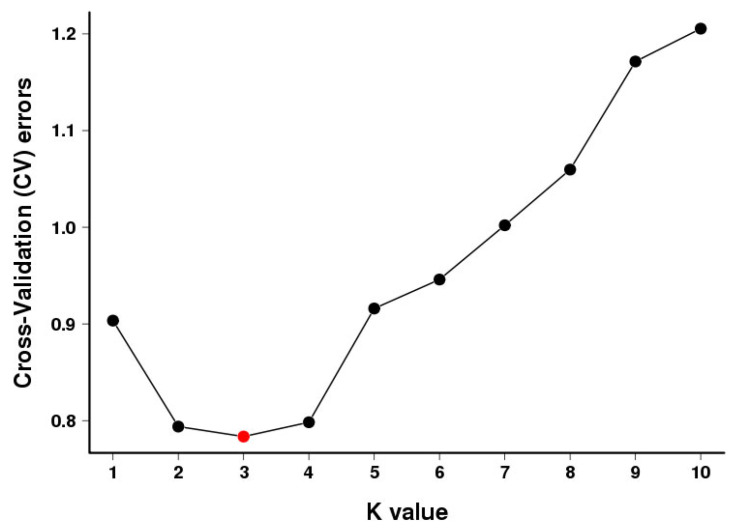
K value cross-validation errors rate.

**Figure 10:**
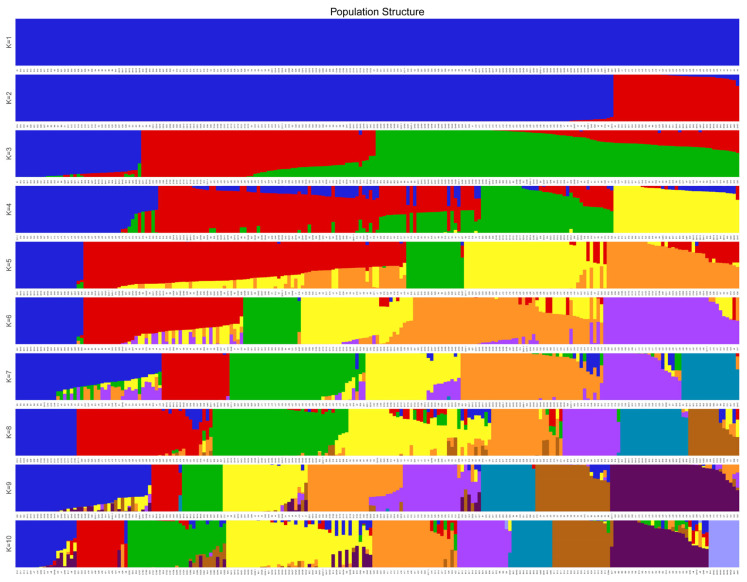
Population structure analysis of 213 *L. ruthenicum* Samples. Different colors represent different subpopulations: blue for Q1, red for Q2, and green for Q3.

Population genetic structure analysis was performed to investigate non-random distribution of genetic variation among the 213 *L. ruthenicum* accessions. Using previously identified SNP markers and Admixture software (version 1.22), we evaluated potential population subdivisions by testing K values (hypothesized ancestral populations) ranging from 1 to 10. The optimal number of genetic clusters (K) was determined primarily based on the minimum cross-validation error rate, as is standard practice. Additionally, we considered the biological interpretability of the clustering results, where K = 3 provided a meaningful representation of the population subdivision that aligned with known geographic origins and breeding history of the germplasm. Cross-validation error rates identified K = 3 as the optimal clustering solution (Figure 9), dividing the accessions into three subgroups (Q1-red, Q2-blue, Q3-green) with distinct genetic backgrounds (Figure 10).

The admixture analysis revealed complex patterns of genetic exchange: Q1 (37 accessions) contained 10 pure ancestral types, with 16, 7, and 4 accessions showing admixture with Q2, Q3, or both, respectively. Q2 (69 accessions) included 31 pure types and 35 admixed with Q3. Q3 (107 accessions) comprised 33 pure types, with extensive admixture (69 accessions) with Q2. Geographic distribution analysis showed Q1 dominated by accessions from Nuomuhong County, Qinghai (21) and Urad Rear Banner, Inner Mongolia (14); Q2 contained materials from Ejin Banner (31), Gansu (13), and Xinjiang (25); while Q3 included accessions from all four provinces (Supplementary Table S2).

Comparative analysis revealed 71.9% concordance (Supplementary Materials Table S1) between population structure (Q1–Q3) and phylogenetic groups (G1–G3), with complete correspondence for Q1–G1 (19 accessions) and Q2-G2 (69 accessions). The dispersion of Inner Mongolian accessions across all three subgroups (Q1–Q3) indicates particularly complex genetic backgrounds in these materials. This comprehensive analysis demonstrates both the overall population differentiation and ongoing genetic exchange among *L. ruthenicum* populations across northwestern China.

### 3.6. PCA of Population Structure

**Figure 11:**
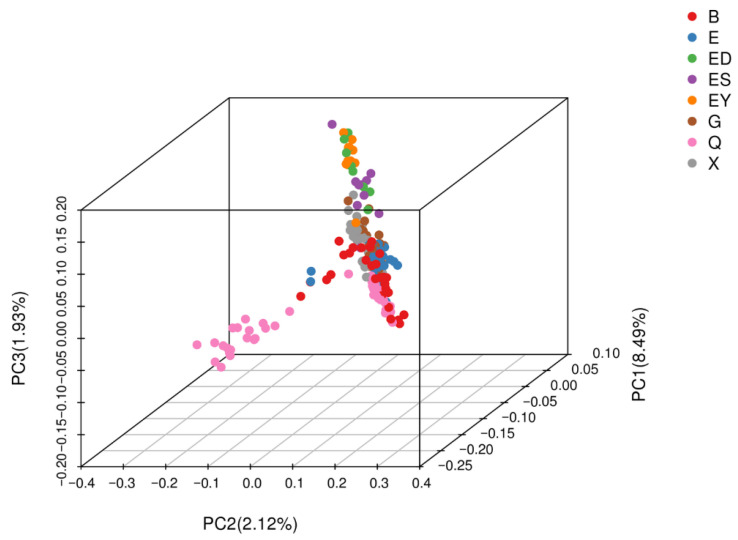
3D clustering plot of Principal component analysis forthe 213 *L. ruthenicum* samples.

**Figure 12:**
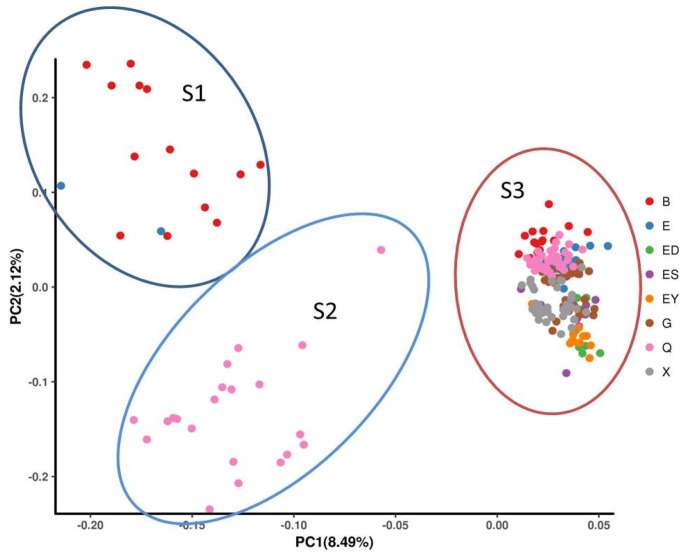
2D Clustering Plot of PCA forthe 213 *L. ruthenicum* samples.

PCA was performed using EIGENSOFT software (version 6.0) to reduce dimensionality and visualize genetic relationships among the 213 *L. ruthenicum* accessions based on SNP variations. The first three principal components, accounting for 8.49%, 2.12%, and 1.93% of total variance, respectively, were selected as covariates to correct population structure. Three-dimensional clustering (Figure 11) and two-dimensional projections (Figure 12) revealed distinct separation along PC1, enabling classification into three groups: S1 (16 accessions), S2 (21 accessions), and S3 (176 accessions).

The PCA results showed partial concordance with previous clustering methods. Group S1 exclusively contained 14 accessions from Urad Rear Banner (Inner Mongolia) and 2 from Dalaihubu Town, while S2 comprised only 21 accessions from Nuomuhong County (Qinghai). The largest cluster S3 incorporated geographically diverse materials (Table 4). Comparative analysis (Table 5) demonstrated that while genetic groups Q1 and phylogenetic clade G1 corresponded to PCA clusters S1/S2, the admixed Q2-Q3 populations collectively formed S3, revealing complementary but non-identical patterns of population subdivision through different analytical approaches.

### 3.7. Analysis of Genetic Diversity

Genetic diversity analysis was conducted to examine intra-specific variation among *L. ruthenicum* populations by assessing differences in allele frequencies and genotype distributions. As shown in Table 6, the population exhibited a mean minor allele frequency (MAF) of 0.235 (range: 0.252–0.296), with expected allele number (Ne) averaging 1.402 (1.31–1.541). The mean expected heterozygosity (He) was 0.235 (0.178–0.318), while Nei’s diversity index (H) averaged 0.253 (0.2–0.327). The population contained an average of 21,088 polymorphic markers (A), with observed allele number (Na) averaging 1.667. Observed heterozygosity (Ho) ranged from 0.054 to 0.092 (mean: 0.075), polymorphism information content (PIC) from 0.142 to 0.255 (mean: 0.188), and Shannon-Wiener index (I) from 0.264 to 0.477 (mean: 0.352).

Notably, accessions from Nuomuhong County, Qinghai Province showed the highest values for all diversity parameters (MAF, Ne, H, A, He, Na, PIC, and I), indicating superior genetic diversity compared to other populations. In contrast, materials from Saihantala Sumu, Ejin Banner, Inner Mongolia exhibited the lowest values for Ne, He, H, A, Na, PIC and I, reflecting reduced genetic diversity in this population. These results demonstrate significant variation in genetic diversity among different geographic sources of *L. ruthenicum*, with implications for conservation and breeding strategies.

### 3.8. Construction of DNA Fingerprints

Following established marker selection criteria [33], we identified 347 high-quality SNP markers for fingerprint development based on stringent parameters including marker quality, representativeness, discriminative power, uniform genomic distribution, and specificity. The selected markers were validated through comprehensive genetic analyses including diversity assessment, phylogenetic reconstruction, population structure examination, and PCA.

**Figure 13:**
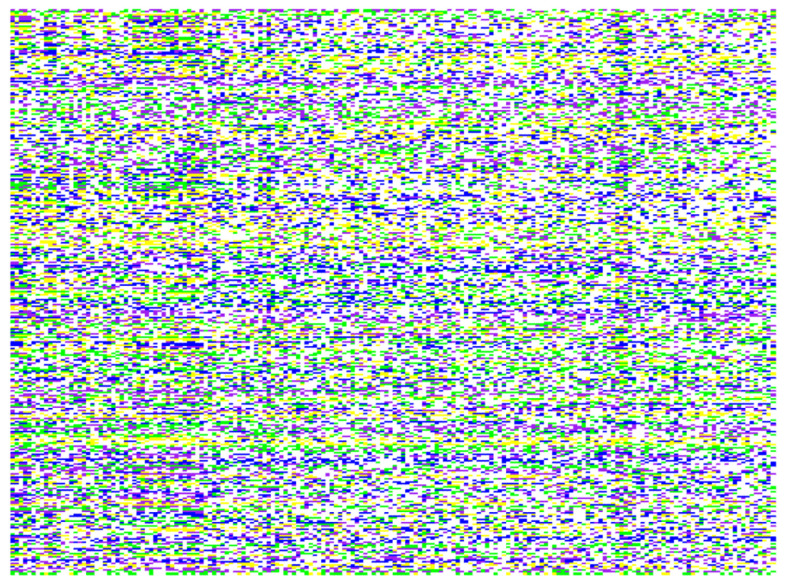
Distribution of candidate markers across samples, with each row representing a filtered candidate locus and each column corresponding to a sample.

For visual representation, we developed two-dimensional barcodes (Figure 13) where nucleotide states were color-coded: yellow (C/C), green (A/A), blue (T/T), and purple (G/G), with gray indicating missing data and white representing heterozygous sites. Using Qrencode software (version 4.1.1), we generated unique 2D barcode fingerprints for each accession, with representative examples shown for six accessions (B1, BH1, E1, G1, Q1, X1) in Figure S1. This fingerprinting system enables rapid, accurate identification of *L. ruthenicum* germplasm while capturing the genetic diversity observed across populations.

## 4. Discussion

### 4.1. SNP-Based Identification of L. ruthenicum Germplasm

The burgeoning market demand for *L. ruthenicum*, a plant with dual medicinal and edible value, has driven rapid expansion of cultivated varieties and intensified germplasm exchange across regions [34]. As a self-incompatible, cross-pollinating species, *L. ruthenicum* exhibits high genomic heterozygosity. Natural selection and hybrid breeding have created complex genetic backgrounds where seed-propagated offspring show significant trait segregation, obscuring varietal relationships [35]. This widespread species demonstrates diverse reproductive strategies, with wild populations evolving into multiple cultivated varieties through domestication and natural genetic variation. Current reliance on morphological identification by experienced practitioners proves inadequate for effective resource utilization, conservation, and breeding programs, underscoring the need for systematic germplasm characterization.

While morphological markers provide preliminary diversity assessments, their environmental sensitivity limits reliability. SSR markers have been widely employed for plant genome mapping, selection, and association studies [36]. Zhao et al. [37] analyzed 139 *L. chinense* accessions using 18 SSR markers, detecting 108 alleles (average 6 per locus) with moderate genetic diversity (gene diversity = 0.3792, PIC = 0.3296) and population structure (three subpopulations, FST = 0.1178), establishing a genetic framework for *L. chinense* improvement. However, SSR markers’ high PCR mutation rates can introduce genotyping errors, complicating multiplex assay development. In contrast, SNPs’ lower mutation rates better suit high-throughput automated detection [7].

SLAF-seq technology enables rapid, cost-effective SNP discovery through restriction-enzyme-based reduced-representation sequencing, permitting comprehensive genome-wide analysis [38]. This approach has successfully identified major QTLs in durum wheat [39], revealed geographical genetic clusters in *Capsicum annuum* [40], and uncovered yield-related loci in bread wheat [41]. Diagnostic SNP development for wheat leaf rust resistance gene *Lr*16 further demonstrates the technique’s utility [42]. Despite these advances, SLAF-seq applications in *L. ruthenicum* genetic studies remained unexplored.

Our SLAF-seq analysis of 213 accessions from eight sources generated 563.48 million reads (606,309–9,355,410 per sample) with excellent quality metrics (average Q30 = 93.32%, GC = 40.11%). We developed 827,630 SLAF tags and identified 1,135,505 SNPs, with 33,121 high-confidence SNPs uniformly distributed across all 10 chromosomes. These results establish a robust foundation for *L. ruthenicum* genetic studies and molecular breeding.

### 4.2. Population Structure of L. Germplasm

Our SLAF-seq analysis of eight geographic populations aimed to facilitate conservation and genetic improvement of *L. ruthenicum* resources. While limited to eight sampling locations, this representative dataset effectively captures the species’ genetic diversity. Phylogenetic analysis revealed three distinct clusters, with Groups 1 and 2 primarily comprising accessions from Urad Rear Banner (Inner Mongolia) and Nuomuhong County (Qinghai), indicating close genetic relationships. Group 3 contained admixed materials from all geographic sources without clear spatial clustering, demonstrating weak genotype-geography correlations.

Population structure analysis (K = 3) showed strong concordance with phylogenetic groupings, while PCA revealed complementary patterns with minor discrepancies, suggesting both narrow genetic bases and distant relatedness among most individuals. The incomplete consistency among analytical methods—with some geographic clusters persisting alongside widespread dispersion—implies complex germplasm exchange history, possibly through seedling transplantation and regional resource sharing. Similar discordances between genetic and geographic patterns were reported in tea plant (Camellia sinensis) populations by Liu et al. [43] through SLAF-seq analysis of 47 Wanzhou tea accessions from Yunnan, Sichuan and Fujian provinces, where clustering analysis revealed three distinct groups that could not be fully classified by geographic origin, and by Fang et al. [44] in Chinese onion germplasm, where population structure (3 groups) and PCA (4 groups) yielded divergent classifications independent of geographic origins. These collective findings highlight the limitations of geographic provenance for predicting genetic relationships in outcrossing perennial crops.

### 4.3. Genetic Diversity of L. ruthenicum Germplasm

The genetic background of *L. ruthenicum* exhibits remarkable complexity, characterized by high genomic heterozygosity and substantial variation, making genetic diversity studies crucial for understanding its genetics, breeding, identification, and evolutionary history [45]. Current research on *Lycium* diversity has primarily relied on phenotypic traits and molecular markers. Yin et al. [19] constructed an SSR-AFLP genetic linkage map in *Lycium* spp., establishing a foundation for QTL mapping and marker-assisted breeding, while Liu et al. [46] employed ISSR and RAMP-PCR markers to reveal genetic connections between geographically distant cultivars, offering valuable insights for medicinal plant conservation. However, these marker systems have shown limitations in fully capturing *Lycium*’s genetic diversity, necessitating more precise methodologies.

Our study represents the first comprehensive analysis of *L. ruthenicum* genetic diversity using SNP markers through SLAF-seq data from 213 accessions. We evaluated diversity using standard metrics including mean allele frequency (0.269), Nei’s diversity index (0.253), polymorphic loci count (21,088), observed alleles (1.666), observed heterozygosity (0.075), polymorphism information content (0.188), and Shannon-Wiener index (0.352). The Nuomuhong population from Qinghai Province consistently showed higher diversity values than other sources, indicating moderate-to-high genetic diversity. Notably, the average PIC value (0.188) classified the germplasm as having low polymorphism (PIC < 0.25), consistent with findings from Guan et al. [47] employing SCoT markers (mean PIC = 0.2253).

Comparative studies reveal intriguing contrasts: Gao et al. [48] reported high diversity in wild *L. barbarum* (He = 0.57, PIC = 0.73) using SSRs, while Zhao et al. [37] documented moderate polymorphism (mean PIC = 0.3296) in *L. chinense*. These discrepancies with our results may stem from methodological differences, as Li et al. [49] found substantial diversity using phenotypic and AFLP analyses. Such variations underscore the importance of integrating multiple approaches and sampling strategies for accurate diversity assessments in *Lycium* species. The observed low average PIC value (0.188) can be attributed to a combination of biological and technical factors. Biologically, the species may have undergone a genetic bottleneck during domestication, reducing overall genetic diversity. Technically, the stringent filtering criteria applied to ensure SNP quality, particularly the minor allele frequency (MAF ≥ 0.05) and locus integrity (INT ≥ 0.3) thresholds, inevitably removed rare alleles from the dataset. While this enhances the reliability of the high-confidence SNP set for population genetic analysis, it can lead to an underestimation of the total polymorphism present in the population, as rare variants contributing to higher PIC values are excluded. Furthermore, the inherent biallelic nature of SNPs imposes a theoretical maximum PIC value lower than that of multi-allelic markers like SSRs, which naturally constrains the upper limit of polymorphism measurable with SNP datasets. The PIC values reported in this study therefore reflect a robust but conservative estimate of genetic diversity, optimized for population genetic inference rather than the absolute capture of all polymorphic sites.

### 4.4. Comparative Analysis of SNP-Based and Phenotypic Clustering in L. ruthenicum Germplasm

High-quality SNPs derived from SLAF-seq data enabled comprehensive phylogenetic, population structure, and principal component analyses, consistently grouping the 213 *L. ruthenicum* accessions into three major clusters. The classification concordance rates reached 71.9% between population structure and phylogenetic groupings, and 60.25% with PCA, demonstrating strong methodological consistency and mutual validation. In contrast, phenotypic trait-based R-type clustering of 31 morphological characteristics divided the accessions into five categories, while Q-type clustering generated six distinct groups, both showing limited concordance with molecular marker-based classifications.

These findings align with Li et al. [49], who reported low consistency between SNP clustering and agronomic trait classifications in sweet orange germplasm. The observed discrepancies highlight that current DNA markers and phenotypic traits lack sufficient correlation for comprehensive germplasm characterization. While molecular markers effectively cluster accessions with shared genetic backgrounds, phenotypic classifications often fail to reflect these relationships due to environmental influences on trait expression. Minor DNA sequence variations undetectable by current marker systems may underlie these phenotypic differences.

Our results underscore the complementary value of integrating molecular markers and phenotypic data—while SNP analyses provide robust genetic relationships, phenotypic evaluations remain essential for validating breeding-relevant characteristics. This dual approach addresses the limitations of each method, as environmental plasticity affects phenotypic expression, and current marker coverage may miss functionally important genomic regions influencing observable traits. The combined strategy offers a more holistic framework for germplasm characterization and breeding program optimization in *L. ruthenicum*.

## 5. Conclusions

This study establishes the first genome-wide high-density SNP marker system for *L. ruthenicum* using SLAF-seq technology, systematically characterizing the genetic diversity and population structure of both wild and cultivated germplasm. Reduced-representation genome sequencing of 213 accessions (average sequencing depth: Q30 > 93%, GC content 40.11%) generated 827,630 SLAF tags and 33,121 high-quality SNPs uniformly distributed across all 12 chromosomes, creating the most comprehensive genomic variation database for this species to date.

Key genetic findings include: (1) Phylogenetic, population structure, and principal component analyses consistently grouped accessions into three genetic clusters with weak geographic correlation (<60% origin consistency), suggesting human-mediated germplasm exchange has reduced isolation-by-distance effects; (2) diversity assessment identified Nuomuhong (Qinghai) populations as having the highest genetic variation (Nei’s index 0.253, Shannon index 0.352), making them priority candidates for core collection development, while the overall low average polymorphism (PIC = 0.183) likely reflects both SNP biallelic limitations and cultivation-induced genetic bottlenecks; (3) the <40% concordance between SNP-based and phenotypic clustering (31 traits) confirms environmental plasticity’s dominant role in trait variation, while highlighting the need for multi-omics integration to decipher complex trait genetics.

These results provide the first genomic SNP reference system for *L. ruthenicum* germplasm identification, with the 33,121 high-confidence markers enabling immediate implementation in marker-assisted breeding. The documented genetic admixture underscores the urgent need for standardized germplasm tracking protocols to conserve wild genetic resources. Future research should integrate transcriptomic and metabolomic data to functionally validate LG10 (SNP hotspot) and elucidate molecular mechanisms underlying key traits like anthocyanin biosynthesis, facilitating the transition from traditional to precision breeding paradigms in *L. ruthenicum* improvement.

## Figures and Tables

**Table 1:** Collection information of germplasm resources.

No.	Seed Source Location	Province	Code	Coordinates	Elevation (m)
1	Urad Rear Banner, Bayannur City	Inner Mongolia	B (B1-29)	E:106.56° N:40.80°	996.0
2	Dalaihubu Town, Ejin Banner	Inner Mongolia	E (E1-29)	E:100.97° N:41.858°	899.6
3	Subonaoer Sumu, Ejin Banner	Inner Mongolia	ES (ES1-10)	E:101.04° N:42.12°	869.2
4	Dongfeng Town, Ejin Banner	Inner Mongolia	ED (ED1-9)	E:101.07° N:42.01°	873.0
5	Saihantala Sumu, Ejin Banner	Inner Mongolia	EY (EY1-11)	E:100.59° N:41.88°	911.0
6	Guazhou County, Gansu	Gansu	G (G1-29)	E:94.99° N:40.26°	1274.0
7	Wushi County, Aksu, Xinjiang	Xinjiang	X (X1-35)	E:78.53° N:41.30°	1379.0
8	Nuomuhong County, Qinghai	Qinghai	Q (Q1-61)	E:96.19° N:36.25°	2712.0

**Table 2:** SLAF number and polymorphic SLAF number in chromosome distribution.

Chromosome ID	SLAF Tag Count	Polymorphic SLAF Tags
Chr01	8537	5074
Chr02	8393	4288
Chr03	7888	4901
Chr04	7542	4625
Chr05	7651	4780
Chr06	7187	4489
Chr07	6799	4212
Chr08	6986	4336
Chr09	6284	3910
Chr10	7150	4515
Chr11	7498	4024
Chr12	6012	4062

**Table 3:** Distribution of the different origin samples in inferred groups clustered by phylogenetic analysis.

Phylogenetic Analysis	B	E	ES	ED	EY	Q	G	X	All	Ratio(%)
G1	14	2	0	0	0	23	0	0	39	18.31
G2	15	0	0	0	0	24	0	0	39	18.31
G3	0	29	9	8	11	14	29	35	135	63.38
All	29	31	9	8	11	61	29	35	213	100%

**Table 4:** Distribution of the different origin samples in inferred groups clustered by population structure.

Population Structure	B	E	ES	ED	EY	Q	G	X	All
Q1	14	1	1	0	0	21	0	0	37
Q2	0	2	9	9	11	0	13	25	69
Q3	14	27	0	0	0	40	16	25	107
All	28	30	10	9	11	61	29	50	213

**Table 5:** Distribution of the different origin samples in inferred groups clustered by PCA.

PCA Groups	B	E	ES	ED	EY	Q	G	X	All
S1	14	2	0	0	0	0	0	0	16
S2	0	0	0	0	0	21	0	0	21
S3	15	26	10	9	11	40	29	50	176
All	20	28	10	9	11	61	29	50	213

**Table 6:** Summary of genetic diversity of the *L. ruthenicum.*

Germplasm Resources	MAF	Ne	He	H	A	Na	Ho	PIC	I
B	0.275	1.000–2.000 (1.506)	0.041–0.500 (0.293)	0.042–0.667 (0.312)	26,302	1.000–2.000 (1.809)	0.038–0.684 (0.075)	0.040–0.375 (0.233)	0.101–0.693 (0.436)
E	0.235	1.000–2.000 (1.432)	0.038–0.500 (0.260)	0.038–0.667 (0.275)	26,622	1.000–2.000 (1.805)	0.038–0.750 (0.070)	0.037–0.375 (0.211)	0.095–0.693 (0.397)
ED	0.296	1.000–2.000 (1.332)	0.105–0.500 (0.191)	0.111–0.667 (0.217)	14,115	1.000–2.000 (1.499)	0.111–1.000 (0.092)	0.099–0.375 (0.151)	0.215–0.693 (0.281)
ES	0.294	1.000–2.000 (1.338)	0.095–0.500 (0.194)	0.100–0.667 (0.220)	14,981	1.000–2.000 (1.509)	0.100–1.000 (0.088)	0.090–0.375 (0.154)	0.199–0.693 (0.286)
EY	0.29	1.000–2.000 (1.310)	0.087–0.500 (0.178)	0.091–0.667 (0.200)	12,732	1.000–2.000 (1.473)	0.091–1.000 (0.078)	0.083–0.375 (0.142)	0.185–0.693 (0.264)
G	0.252	1.000–2.000 (1.387)	0.036–0.500 (0.229)	0.037–0.667 (0.241)	22,279	1.000–2.000 (1.673)	0.037–0.731 (0.069)	0.036–0.375 (0.184)	0.092–0.693 (0.345)
Q	0.256	1.000–2.000 (1.541)	0.024–0.500 (0.318)	0.024–0.556 (0.327)	30,640	1.000–2.000 (1.925)	0.023–0.595 (0.070)	0.024–0.375 (0.255)	0.066–0.693 (0.477)
X	0.255	1.000–2.000 (1.371)	0.033–0.500 (0.219)	0.033–0.667 (0.232)	21,040	1.000–2.000 (1.638)	0.033–0.667 (0.054)	0.032–0.375 (0.176)	0.085–0.693 (0.329)
Mean	0.269	1.402	0.235	0.253	21,089	1.667	0.075	0.188	0.352

Numbers before and in brackets indicate range and mean, respectively. MAF, Minor allele frequency; Ne, Expected number of alleles; He, Expected heterozygosity; H, Nei’s genetic diversity index; A, Number of polymorphic loci; Na, Observed number of alleles; Ho, Observed heterozygosity; PIC, Polymorphism information content; I, Shannon’s Wiener index.

## Data Availability

The original contributions presented in this study are included in the article/Supplementary Materials. Further inquiries can be directed to the corresponding authors.

## Supplementary Materials

The following supporting information can be downloaded at: https://www.mdpi.com/article/10.3390/plants14213374/s1, Figure S1: QR code fingerprint spectrum of representative *L. ruthenicum* accessions. Table S1: The genetic groups among 213 *Lycium ruthenicum* Murr. samples by population structure analysis. Table S2: 213 *L. ruthenicum* samples on population structure analysis, phylogenetic analysis and PCA.
