# Supplementary figures and images for: Genome-Wide SNP Analysis Reveals Population Structure and Genetic Diversity in Lycium ruthenicum Murr
Source: Plants (Basel). 2025 Nov 4;14(21):3374. doi: 10.3390/plants14213374 (PMC12610066; doi:10.3390/plants14213374)

## Slide 1
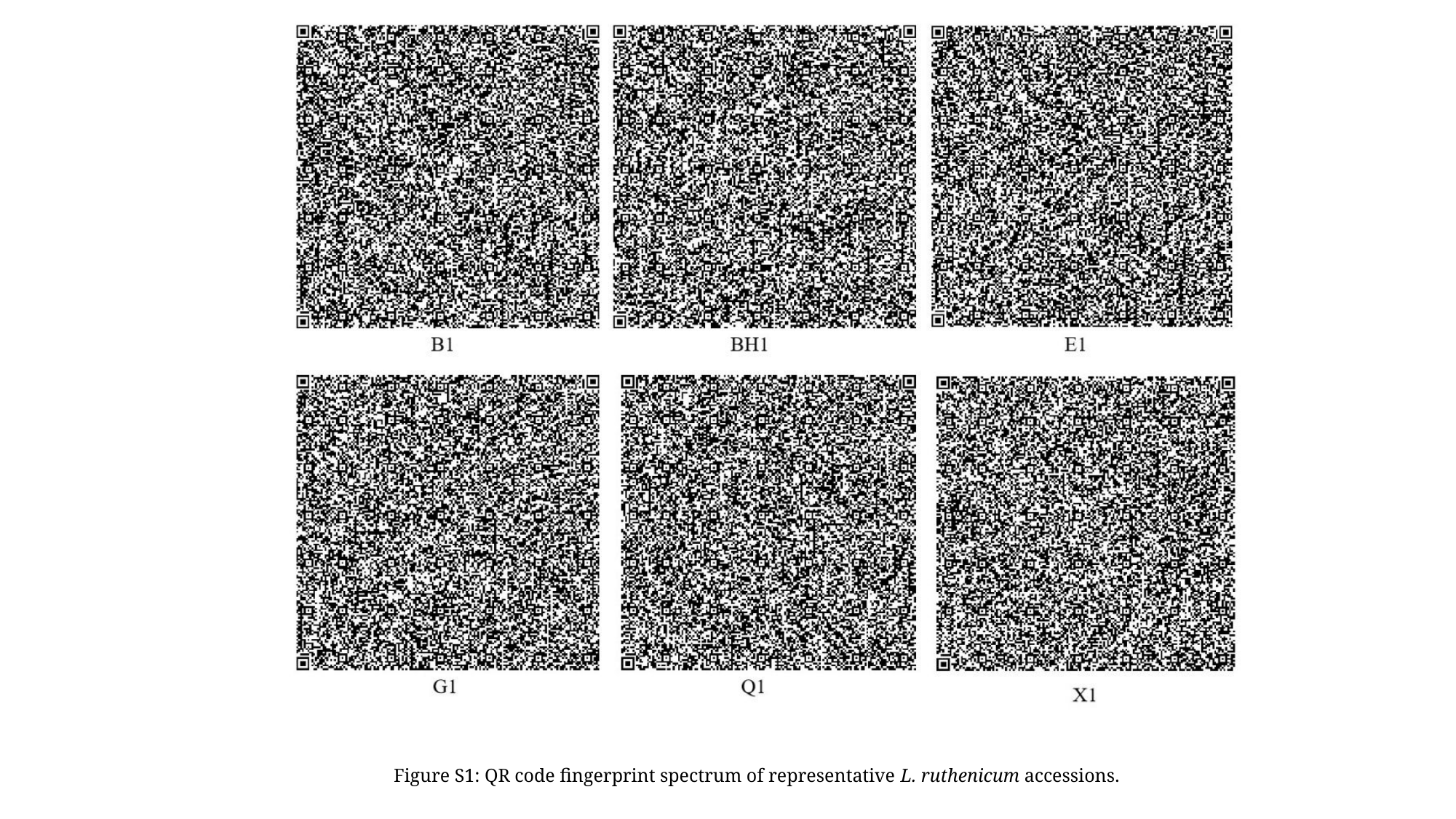

Figure S1: QR code fingerprint spectrum of representative L. ruthenicum accessions.

Supplement: Supplementary file 1 [file plants-14-03374-s001.zip › plants-3920846-supplementary/Figure S1.pptx]
